# Crystal structure of 3-*C*-(*N*-benzyl­oxy­carbon­yl)amino­methyl-3-de­oxy-1,2:5,6-di-*O*-iso­propyl­idene-α-d-allo­furan­ose

**DOI:** 10.1107/S2056989015017582

**Published:** 2015-09-26

**Authors:** Vitalijs Rjabovs, Dmitrijs Stepanovs, Maris Turks

**Affiliations:** a Institute of Technology of Organic Chemistry, Faculty of Materials Science and Applied Chemistry, Riga Technical University, P. Valdena 3/7, Riga, LV-1048, Latvia; b Latvian Institute of Organic Synthesis, Aizkraukles 21, Riga, LV-1006, Latvia

**Keywords:** crystal structure, 3-amino­methyl diacetone-d-allose, imino sugar precursor, sugar amino acid precursor, hydrogen bonding.

## Abstract

The title compound, C_21_H_29_NO_7_ (**1**) [systematic name: benzyl ({(3a*R*,5*S*,6*R*,6a*R*)-5-[(*R*)-2,2-dimethyl-1,3-dioxolan-4-yl]-2,2-di­methyl­tetrahydro­furo[2,3-*d*][1,3]dioxol-6-yl}meth­yl)carbamate], consists of a substituted 2,2-di­methyl­tetra­hydro­furo[2,3-*d*][1,3]dioxolane skeleton. The furan­ose ring adopts an envelope conformation close to *C*_3_-*exo*, where the C atom substituted by the benzyl carbamate group is the flap. The fused dioxolane ring also adopts an envelope conformation, as does the terminal dioxolane ring, with in each case an O atom as the flap. In the crystal, mol­ecules are linked by N—H⋯O and C–H⋯O hydrogen bonds, forming chains propagating along the *b*-axis direction.

## Abstract 2

The title compound consists of a substituted 2,2-di­methyl­tetra­hydro­furo[2,3-*d*][1,3]dioxolane skeleton. The furan­ose ring adopts a conformation close to *C*_3_-*exo*. Both dioxolane rings adopt envelope conformations with an O atom as the flap in each case. In the crystal, mol­ecules are linked by N—H⋯O hydrogen bonds, forming chains propagating along the *b*-axis direction.

## Chemical context   

**Figure 1:**
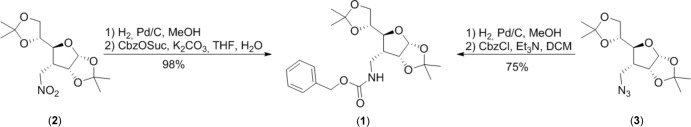
Synthesis of the title compound.

The title compound, 3-*C*-(*N*-benzyl­oxycarbon­yl)amino­methyl-3-de­oxy-1,2:5,6-di-*O*-iso­propyl­idene-α-d-allo­furan­ose (**1**), was obtained as an inter­mediate in the syntheses of carbohydrate-based non-natural amino acids, so called sugar amino acids (Rjabovs *et al.*, 2015 ▸), by hydrogenation and carbamate protection of either nitro (Lugiņina *et al.*, 2013 ▸) or azido (Filichev & Pedersen, 2001 ▸; Rjabova *et al.*, 2012 ▸) precursors (Fig. 1 ▸).
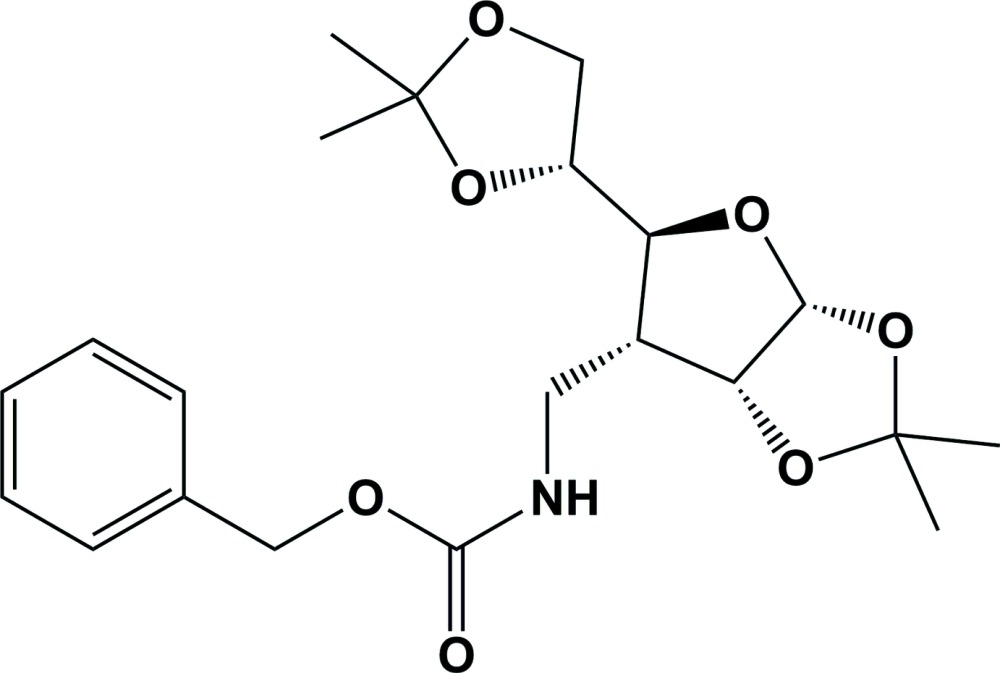


The synthesis of sugar amino acids and their properties and applications have been reported on by Rjabovs *et al.* (2015 ▸), and reviewed by Rjabovs & Turks (2013 ▸) and Risseeuw *et al.* (2013 ▸). The title compound can be used as a precursor for the syntheses of imino sugars and 10-aza-*C*-nucleosides (Filichev & Pedersen, 2001 ▸). The syntheses and biological properties of imino sugars have been reviewed by López *et al.* (2012 ▸), while the syntheses and biological properties of aza-nucleosides have been reported on by Romeo *et al.* (2010 ▸) and Merino (2006 ▸).

## Structural commentary   

**Figure 2:**
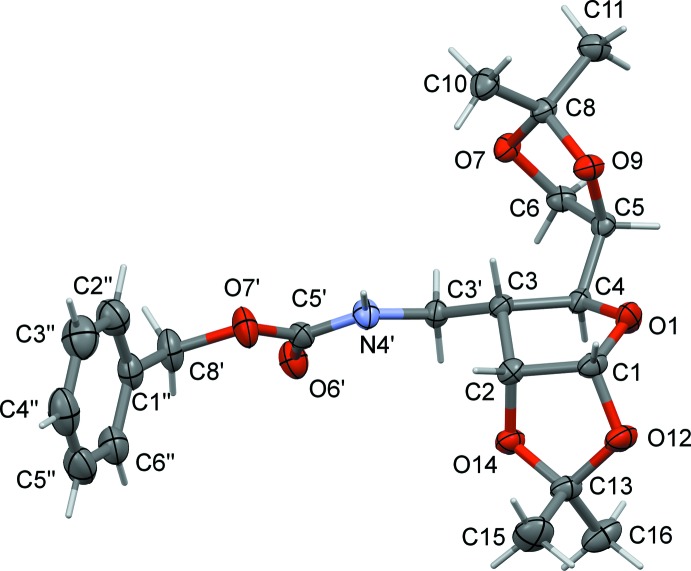
The mol­ecular structure of compound (**1**), with atom labelling. Displacement ellipsoids are drawn at the 50% probability level.

The title compound, Fig. 2 ▸, consists of a tetra­hydro­furan core fused with a dioxolane ring and substituted with dioxolane and (*N*-benzyl­oxycarbon­yl)amino­methyl moieties. The furan­ose ring adopts a conformation close to *C*_3_-*exo*. On the other hand, the furan­ose ring may be viewed as an envelope, where atom C3 deviates from the mean plane through atoms O1/C1/C2/C4 by 0.567 (2) Å. The fused dioxolane ring also adopts an envelope conformation, where O14 deviates from the mean plane through the four near planar atoms (O12/C1/C2/C13) by 0.422 (2) Å. The dihedral angle between the planar fragments of these rings is 67.1 (1)°. The five-membered ring of the 2,2-dimethyl-1,3-dioxolan-4-yl group also adopts an envelope conformation, with atom O7 deviating from the mean plane through the four planar atoms (O9/C5/C6/C8) by 0.519 (1) Å.

## Supra­molecular features   

**Figure 3:**
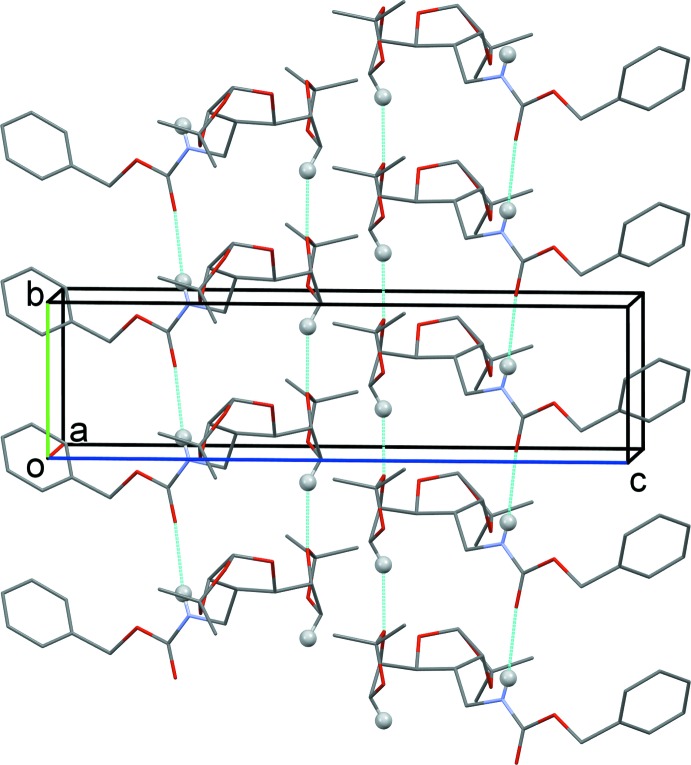
The crystal packing of compound (**1**), viewed along the *a* axis. Hydrogen bonds are shown as dashed lines (see Table 1 ▸ for details). For clarity only H atoms involved in these inter­actions have been included.

In the crystal, mol­ecules are linked by N—H⋯O and C–H⋯O hydrogen bonds, forming chains propagating along the *b*-axis direction (Fig. 3 ▸ and Table 1 ▸).

## Database survey   

A search of the Cambridge Structural Database (Version 5.36; Groom & Allen, 2014 ▸) for substituted 3a,5,6,6a-tetra­hydro­furo[2,3-*d*][1,3]dioxoles gave 485 hits (excluding metal-org­anics). However, only two structures are 3a,5,6,6a-tetra­hydrofuro[2,3-*d*][1,3]dioxol-6-yl­methyl­carbamic acid derivatives, *viz*. (3*R*)-3′-ethyl-1,2:5,6-di-*O*-iso­propyl­idene-spiro­(3-de­oxy-*a*-d-allo­furan­ose-3,5′-oxazolidin)-2′-one (CIDVES; Turks *et al.*, 2013 ▸), and (3*R*)-3′-phenyl­acetyl-1,2:5,6-di-*O*-iso­propylidene­spiro­(3-de­oxy-*a*-d-allo­furan­ose-3,5′-oxazolidin)-2′-one (YIMBED; Turks *et al.*, 2013 ▸).

## Synthesis and crystallization   

The two methods for the synthesis of compound (**1**) are illus­trated in Fig. 1 ▸.

**From compound (2)**: A mixture of nitro­methyl compound (**2**) (5.00 g, 16.5 mmol, 1 equiv.) and 10% Pd/C (1.00 g) in MeOH (200 ml) was hydrogenated under 40 atm pressure at 313 K overnight (TLC control). The resulting reaction mixture was filtered through celite and the filtrate was evaporated under reduced pressure. The residue was dissolved in THF (60 ml) and a solution of K_2_CO_3_ (2.50 g, 18.1 mmol, 1.1 equiv.) in water (35 ml) was added. The resulting mixture was cooled to 273 K and *N*-(benzyl­oxycarbon­yloxy)succinimide (4.50 g, 18.1 mmol, 1.1 equiv) was added portion-wise. The reaction mixture was stirred at 273 K for 4 h (TLC control). Solid K_2_CO_3_ (1 g) was added and the formed layers were separated. The organic phase was washed with saturated aqueous solution of NaHSO_4_ (50 ml) while the aqueous phase was extracted with a mixture of hexa­nes and CH_2_Cl_2_ (3 × 100 ml, 8:2 *v*/*v*). The combined organic phase was washed with brine (2 × 100 ml), dried over Na_2_SO_4_, filtered and evaporated under reduced pressure. Crude product (**1**) was obtained as a yellow oil (6.60 g, 98% crude) and used further without additional purification.

**From compound (3)**: Through a mixture of azide (**3**) (14.86 g, 49.7 mmol, 1.0 equiv) and 10% Pd/C (1.45 g) in MeOH (150 ml) hydrogen flow was passed at ambient temperature and pressure for 1 h (TLC control). The reaction mixture was filtered through a celite pad and the filtrate was evaporated under reduced pressure. The residue was dissolved in anhydrous CH_2_Cl_2_ (200 ml) and tri­ethyl­amine (8.5 ml, 61.0 mmol, 1.0 equiv) was added. The resulting solution was cooled to 273 K and benzyl chloro­formate (7.0 ml, 60.5 mmol, 1.2 equiv) was added portion-wise. The reaction mixture was stirred under an argon atmosphere at ambient temperature overnight. The solvent was evaporated under reduced pressure and the residue was dissolved in EtOAc (100 ml). The resulting solution was washed with a saturated aqueous solution of NaHCO_3_ (3 × 20 ml) and brine (3 × 30 ml), dried over Na_2_SO_4_, filtered and evaporated. Column chromatography (hexa­nes/EtOAc 4:1 to 2:1 *v*/*v*) yielded product (**1**) (15.22 g, 75%) as a colourless oil that solidifies at low temperatures. *R*_f_ = 0.6 (hexa­nes/EtOAc 1:1). ^1^H NMR (CDCl_3_, 300 MHz): 1.30, 1.34, 1.41, 1.50 (4*s*, 12H, 2 (H_3_C)_2_C), 2.13 [*dq*, *J* = 9.6, 4.9 Hz, 1H, H-C(3)], 3.52 [*m*, 2H, H_2_C(3′)], 3.77 [*m*, 1H, H-C(5)], 3.95 [*m*, 2H, H_2_C(6)], 4.11 [*m*, 1H, H-C(4]), 4.68 [*t*, *J* = 4.3 Hz, 1H, H-C(2)], 5.11 (*s*, AB syst., 2H, H_2_C-Ph), 5.67 (*t*, *J* = 6.0 Hz, 1H, HN), 5.75 [*d*, *J* = 3.8 Hz, 1H, H-C(1)], 7.35 (*m*, 5H, Ph). ^13^C NMR (CDCl_3_, 75 MHz): 25.2, 26.3, 26.5, 26.7, 38.0, 48.6, 66.5, 67.8, 77.3, 81.4, 82.0, 104.8, 109.8, 112.2, 128.0, 128.0, 128.5, 136.8, 156.4. HRMS: Calculated for C_21_H_29_NO_7_Na, [*M* + Na]^+^ 430.1842. Found: 430.1795.

X-ray quality single crystals were obtained by spontaneous crystallization of the title compound from the neat oily material at 277 K.

## Refinement   

Crystal data, data collection and structure refinement details are summarized in Table 2 ▸. The H atom on the amino group was located in a difference Fourier map and freely refined. The C-bound H atoms were positioned geometrically and refined as riding on their parent atoms: C—H = 0.93–0.98Å with *U*_iso_(H) = 1.5*U*_eq_(C) for methyl H atoms and 1.2*U*_eq_(C) for other H atoms. Reflections (1,0,0) and (0,0,2), whose intensities were affected by the beam-stop, were removed from the final refinement.

## Supplementary Material

Crystal structure: contains datablock(s) I. DOI: 10.1107/S2056989015017582/su5210sup1.cif

Structure factors: contains datablock(s) I. DOI: 10.1107/S2056989015017582/su5210Isup2.hkl

CCDC reference: 1425954

Additional supporting information:  crystallographic information; 3D view; checkCIF report

## Figures and Tables

**Table 1:** Hydrogen-bond geometry (, )

*D*H*A*	*D*H	H*A*	*D* *A*	*D*H*A*
N4H4O6^i^	0.80(3)	2.51(3)	3.295(3)	167(2)
C6H6*B*O9^ii^	0.97	2.32	3.184(3)	141

Symmetry codes: (i) 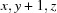; (ii) 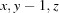.

**Table 2:** Experimental details

Crystal data	
Chemical formula	C_21_H_29_NO_7_
*M*_r_	407.45
Crystal system, space group	Monoclinic, *P*2_1_
Temperature (K)	173
*a*, *b*, *c* ()	9.3235(3), 5.4118(1), 20.4381(7)
()	96.748(1)
*V* (^3^)	1024.10(5)
*Z*	2
Radiation type	Mo *K*
(mm^1^)	0.10
Crystal size (mm)	0.32 0.31 0.20

Data collection	
Diffractometer	Nonius KappaCCD
No. of measured, independent and observed [*I* > 2(*I*)] reflections	5535, 3279, 2597
*R*_int_	0.034
(sin /)_max_ (^1^)	0.705

Refinement	
*R*[*F* ^2^ > 2(*F* ^2^)], *wR*(*F* ^2^), *S*	0.045, 0.100, 1.03
No. of reflections	3279
No. of parameters	270
No. of restraints	1
H-atom treatment	H atoms treated by a mixture of independent and constrained refinement
_max_, _min_ (e ^3^)	0.23, 0.21

Computer programs: *KappaCCD Server Software* (Nonius, 1997 ▸), *HKL* *DENZO* and *SCALEPACK* (Otwinowski Minor, 1997 ▸), *SIR2011* (Burla *et al.*, 2012 ▸), *Mercury* (Macrae *et al.*, 2008 ▸), *SHELXL97* (Sheldrick, 2008 ▸), *PLATON* (Spek, 2009 ▸) and *publCIF* (Westrip, 2010 ▸).

## supplementary crystallographic information

### Crystal data

**Table:**

C_21_H_29_NO_7_	*F*(000) = 436
*M**_r_* = 407.45	*D*_x_ = 1.321 Mg m^−^^3^
Monoclinic, *P*2_1_	Mo *K*α radiation, λ = 0.71073 Å
Hall symbol: P 2yb	Cell parameters from 11906 reflections
*a* = 9.3235 (3) Å	θ = 1.0–30.0°
*b* = 5.4118 (1) Å	µ = 0.10 mm^−^^1^
*c* = 20.4381 (7) Å	*T* = 173 K
β = 96.748 (1)°	Block, colourless
*V* = 1024.10 (5) Å^3^	0.32 × 0.31 × 0.20 mm
*Z* = 2	

### Data collection

**Table:**

Nonius KappaCCD diffractometer	2597 reflections with *I* > 2σ(*I*)
Radiation source: fine-focus sealed tube	*R*_int_ = 0.034
Graphite monochromator	θ_max_ = 30.1°, θ_min_ = 2.3°
CCD scans	*h* = −13→13
5535 measured reflections	*k* = −7→6
3279 independent reflections	*l* = −28→28

### Refinement

**Table:**

Refinement on *F*^2^	Primary atom site location: structure-invariant direct methods
Least-squares matrix: full	Secondary atom site location: difference Fourier map
*R*[*F*^2^ > 2σ(*F*^2^)] = 0.045	Hydrogen site location: inferred from neighbouring sites
*wR*(*F*^2^) = 0.100	H atoms treated by a mixture of independent and constrained refinement
*S* = 1.03	*w* = 1/[σ^2^(*F*_o_^2^) + (0.044*P*)^2^ + 0.1203*P*] where *P* = (*F*_o_^2^ + 2*F*_c_^2^)/3
3279 reflections	(Δ/σ)_max_ < 0.001
270 parameters	Δρ_max_ = 0.23 e Å^−^^3^
1 restraint	Δρ_min_ = −0.21 e Å^−^^3^

### Special details

**Table:**

Geometry. All e.s.d.'s (except the e.s.d. in the dihedral angle between two l.s. planes) are estimated using the full covariance matrix. The cell e.s.d.'s are taken into account individually in the estimation of e.s.d.'s in distances, angles and torsion angles; correlations between e.s.d.'s in cell parameters are only used when they are defined by crystal symmetry. An approximate (isotropic) treatment of cell e.s.d.'s is used for estimating e.s.d.'s involving l.s. planes.
Refinement. Refinement of *F*^2^ against ALL reflections. The weighted *R*-factor *wR* and goodness of fit *S* are based on *F*^2^, conventional *R*-factors *R* are based on *F*, with *F* set to zero for negative *F*^2^. The threshold expression of *F*^2^ > σ(*F*^2^) is used only for calculating *R*-factors(gt) *etc*. and is not relevant to the choice of reflections for refinement. *R*-factors based on *F*^2^ are statistically about twice as large as those based on *F*, and *R*- factors based on ALL data will be even larger.

### Fractional atomic coordinates and isotropic or equivalent isotropic displacement parameters (Å^2^)

**Table:**

	*x*	*y*	*z*	*U*_iso_*/*U*_eq_	
O1	0.34514 (16)	0.3163 (3)	0.37758 (7)	0.0315 (4)	
C1	0.2837 (2)	0.3609 (4)	0.31216 (10)	0.0257 (4)	
H1	0.2922	0.5356	0.3006	0.031*	
C2	0.3645 (2)	0.1973 (4)	0.26790 (10)	0.0236 (4)	
H2	0.3840	0.2807	0.2273	0.028*	
C3	0.5014 (2)	0.1255 (4)	0.31153 (9)	0.0216 (4)	
H3	0.5695	0.2635	0.3118	0.026*	
C4	0.4468 (2)	0.1149 (4)	0.37946 (9)	0.0219 (4)	
H4	0.3965	−0.0419	0.3842	0.026*	
C5	0.5571 (2)	0.1572 (4)	0.43934 (10)	0.0221 (4)	
H5	0.5063	0.1803	0.4782	0.027*	
C6	0.6694 (2)	−0.0473 (4)	0.45328 (10)	0.0239 (4)	
H6A	0.6898	−0.0777	0.5002	0.029*	
H6B	0.6366	−0.1997	0.4314	0.029*	
O7	0.79329 (15)	0.0472 (3)	0.42712 (7)	0.0251 (3)	
C8	0.7920 (2)	0.3060 (4)	0.43977 (10)	0.0234 (4)	
O9	0.64107 (15)	0.3721 (3)	0.43018 (7)	0.0251 (3)	
C10	0.8696 (3)	0.4342 (4)	0.38914 (12)	0.0326 (5)	
H10A	0.8217	0.3997	0.3459	0.049*	
H10B	0.9674	0.3758	0.3923	0.049*	
H10C	0.8695	0.6092	0.3968	0.049*	
C11	0.8532 (2)	0.3653 (4)	0.51018 (11)	0.0313 (5)	
H11A	0.9549	0.3308	0.5161	0.047*	
H11B	0.8057	0.2657	0.5400	0.047*	
H11C	0.8376	0.5369	0.5190	0.047*	
O12	0.13924 (16)	0.2825 (3)	0.30147 (9)	0.0356 (4)	
C13	0.1257 (2)	0.0752 (4)	0.25826 (11)	0.0277 (5)	
O14	0.27047 (15)	−0.0114 (3)	0.25586 (7)	0.0282 (3)	
C15	0.0589 (3)	0.1589 (6)	0.19055 (13)	0.0490 (7)	
H15A	0.1160	0.2897	0.1752	0.074*	
H15B	−0.0375	0.2176	0.1932	0.074*	
H15C	0.0558	0.0225	0.1604	0.074*	
C16	0.0396 (3)	−0.1209 (5)	0.28758 (14)	0.0411 (6)	
H16A	−0.0564	−0.0612	0.2904	0.062*	
H16B	0.0849	−0.1617	0.3309	0.062*	
H16C	0.0352	−0.2656	0.2602	0.062*	
C3'	0.5763 (2)	−0.1049 (4)	0.29004 (10)	0.0240 (4)	
H3'1	0.6569	−0.1470	0.3226	0.029*	
H3'2	0.5093	−0.2427	0.2863	0.029*	
N4'	0.6281 (2)	−0.0585 (4)	0.22661 (9)	0.0279 (4)	
C5'	0.6706 (2)	−0.2436 (4)	0.18935 (10)	0.0279 (5)	
O6'	0.66529 (19)	−0.4616 (3)	0.20170 (8)	0.0363 (4)	
O7'	0.71820 (19)	−0.1464 (3)	0.13471 (8)	0.0403 (4)	
C8'	0.7650 (3)	−0.3172 (5)	0.08682 (11)	0.0418 (6)	
H8'1	0.7087	−0.4682	0.0856	0.050*	
H8'2	0.8662	−0.3584	0.0979	0.050*	
C1''	0.7418 (3)	−0.1870 (5)	0.02154 (11)	0.0355 (5)	
C2''	0.8230 (3)	0.0185 (6)	0.00934 (12)	0.0444 (6)	
H2''	0.8968	0.0708	0.0409	0.053*	
C3''	0.7954 (3)	0.1460 (6)	−0.04903 (14)	0.0525 (7)	
H3''	0.8502	0.2845	−0.0565	0.063*	
C4''	0.6868 (3)	0.0694 (6)	−0.09655 (13)	0.0520 (8)	
H4''	0.6679	0.1563	−0.1359	0.062*	
C5''	0.6074 (3)	−0.1351 (7)	−0.08519 (13)	0.0523 (8)	
H5''	0.5349	−0.1882	−0.1173	0.063*	
C6''	0.6334 (3)	−0.2644 (6)	−0.02641 (13)	0.0454 (7)	
H6''	0.5783	−0.4028	−0.0191	0.055*	
H4'	0.647 (3)	0.080 (5)	0.2161 (12)	0.028 (7)*	

### Atomic displacement parameters (Å^2^)

**Table:**

	*U*^11^	*U*^22^	*U*^33^	*U*^12^	*U*^13^	*U*^23^
O1	0.0247 (8)	0.0388 (9)	0.0308 (8)	0.0114 (7)	0.0028 (6)	−0.0044 (7)
C1	0.0195 (10)	0.0230 (9)	0.0345 (11)	0.0010 (8)	0.0020 (8)	0.0002 (9)
C2	0.0211 (10)	0.0238 (10)	0.0262 (10)	−0.0003 (8)	0.0038 (8)	0.0025 (8)
C3	0.0170 (9)	0.0232 (9)	0.0249 (10)	−0.0002 (8)	0.0032 (7)	−0.0002 (8)
C4	0.0189 (9)	0.0221 (10)	0.0255 (10)	0.0005 (7)	0.0065 (8)	−0.0007 (8)
C5	0.0225 (10)	0.0197 (9)	0.0250 (10)	−0.0009 (9)	0.0062 (8)	−0.0007 (8)
C6	0.0250 (11)	0.0190 (9)	0.0272 (10)	−0.0019 (8)	0.0016 (8)	0.0025 (8)
O7	0.0232 (7)	0.0175 (6)	0.0356 (8)	0.0016 (6)	0.0072 (6)	0.0001 (6)
C8	0.0191 (10)	0.0202 (9)	0.0309 (11)	−0.0007 (8)	0.0035 (8)	−0.0022 (8)
O9	0.0209 (7)	0.0162 (6)	0.0373 (8)	0.0016 (6)	−0.0002 (6)	−0.0009 (6)
C10	0.0311 (12)	0.0280 (11)	0.0403 (13)	−0.0024 (10)	0.0107 (9)	−0.0010 (10)
C11	0.0267 (11)	0.0284 (10)	0.0373 (12)	0.0007 (9)	−0.0031 (9)	−0.0035 (10)
O12	0.0190 (7)	0.0322 (9)	0.0553 (10)	0.0009 (7)	0.0031 (7)	−0.0107 (8)
C13	0.0202 (10)	0.0256 (10)	0.0361 (12)	0.0009 (8)	−0.0015 (8)	−0.0003 (9)
O14	0.0206 (7)	0.0272 (8)	0.0361 (8)	0.0014 (6)	0.0002 (6)	−0.0051 (6)
C15	0.0341 (13)	0.0673 (18)	0.0430 (15)	0.0070 (15)	−0.0067 (11)	0.0092 (14)
C16	0.0270 (12)	0.0316 (12)	0.0651 (17)	0.0012 (10)	0.0069 (11)	0.0080 (12)
C3'	0.0235 (10)	0.0266 (10)	0.0226 (10)	0.0031 (8)	0.0053 (8)	−0.0002 (8)
N4'	0.0324 (10)	0.0264 (9)	0.0262 (9)	−0.0010 (8)	0.0086 (7)	−0.0008 (8)
C5'	0.0249 (11)	0.0371 (12)	0.0215 (10)	0.0018 (9)	0.0015 (8)	−0.0028 (9)
O6'	0.0486 (11)	0.0295 (8)	0.0319 (9)	0.0045 (8)	0.0087 (7)	−0.0024 (7)
O7'	0.0574 (11)	0.0382 (9)	0.0287 (8)	0.0009 (9)	0.0195 (7)	−0.0046 (7)
C8'	0.0536 (15)	0.0439 (14)	0.0300 (12)	0.0132 (13)	0.0142 (11)	−0.0045 (11)
C1''	0.0417 (13)	0.0390 (13)	0.0274 (11)	0.0092 (11)	0.0106 (9)	−0.0045 (10)
C2''	0.0479 (15)	0.0494 (15)	0.0354 (14)	0.0047 (13)	0.0027 (11)	−0.0031 (12)
C3''	0.0637 (18)	0.0468 (15)	0.0491 (17)	0.0009 (16)	0.0156 (14)	0.0045 (14)
C4''	0.0669 (19)	0.0580 (19)	0.0323 (14)	0.0206 (16)	0.0110 (13)	0.0033 (13)
C5''	0.0498 (16)	0.075 (2)	0.0310 (13)	0.0108 (17)	0.0005 (11)	−0.0100 (14)
C6''	0.0480 (15)	0.0546 (16)	0.0352 (13)	0.0027 (13)	0.0111 (11)	−0.0086 (12)

### Geometric parameters (Å, º)

**Table:**

O1—C1	1.412 (2)	C13—C16	1.498 (3)
O1—C4	1.442 (2)	C13—C15	1.519 (3)
C1—O12	1.404 (2)	C15—H15A	0.9600
C1—C2	1.526 (3)	C15—H15B	0.9600
C1—H1	0.9800	C15—H15C	0.9600
C2—O14	1.433 (2)	C16—H16A	0.9600
C2—C3	1.519 (3)	C16—H16B	0.9600
C2—H2	0.9800	C16—H16C	0.9600
C3—C3'	1.519 (3)	C3'—N4'	1.457 (3)
C3—C4	1.535 (3)	C3'—H3'1	0.9700
C3—H3	0.9800	C3'—H3'2	0.9700
C4—C5	1.520 (3)	N4'—C5'	1.346 (3)
C4—H4	0.9800	N4'—H4'	0.80 (3)
C5—O9	1.427 (2)	C5'—O6'	1.209 (3)
C5—C6	1.527 (3)	C5'—O7'	1.355 (3)
C5—H5	0.9800	O7'—C8'	1.450 (3)
C6—O7	1.424 (3)	C8'—C1''	1.502 (3)
C6—H6A	0.9700	C8'—H8'1	0.9700
C6—H6B	0.9700	C8'—H8'2	0.9700
O7—C8	1.424 (2)	C1''—C2''	1.384 (4)
C8—O9	1.443 (2)	C1''—C6''	1.387 (4)
C8—C10	1.501 (3)	C2''—C3''	1.376 (4)
C8—C11	1.519 (3)	C2''—H2''	0.9300
C10—H10A	0.9600	C3''—C4''	1.382 (4)
C10—H10B	0.9600	C3''—H3''	0.9300
C10—H10C	0.9600	C4''—C5''	1.367 (5)
C11—H11A	0.9600	C4''—H4''	0.9300
C11—H11B	0.9600	C5''—C6''	1.387 (4)
C11—H11C	0.9600	C5''—H5''	0.9300
O12—C13	1.424 (3)	C6''—H6''	0.9300
C13—O14	1.435 (3)		
			
C1—O1—C4	110.30 (15)	C1—O12—C13	110.36 (16)
O12—C1—O1	111.76 (17)	O12—C13—O14	105.28 (15)
O12—C1—C2	105.29 (17)	O12—C13—C16	108.82 (19)
O1—C1—C2	106.75 (16)	O14—C13—C16	109.43 (18)
O12—C1—H1	110.9	O12—C13—C15	109.1 (2)
O1—C1—H1	110.9	O14—C13—C15	110.69 (19)
C2—C1—H1	110.9	C16—C13—C15	113.2 (2)
O14—C2—C3	110.74 (16)	C2—O14—C13	107.18 (15)
O14—C2—C1	102.96 (16)	C13—C15—H15A	109.5
C3—C2—C1	103.87 (16)	C13—C15—H15B	109.5
O14—C2—H2	112.8	H15A—C15—H15B	109.5
C3—C2—H2	112.8	C13—C15—H15C	109.5
C1—C2—H2	112.8	H15A—C15—H15C	109.5
C2—C3—C3'	115.04 (17)	H15B—C15—H15C	109.5
C2—C3—C4	101.29 (16)	C13—C16—H16A	109.5
C3'—C3—C4	116.30 (17)	C13—C16—H16B	109.5
C2—C3—H3	107.9	H16A—C16—H16B	109.5
C3'—C3—H3	107.9	C13—C16—H16C	109.5
C4—C3—H3	107.9	H16A—C16—H16C	109.5
O1—C4—C5	106.73 (15)	H16B—C16—H16C	109.5
O1—C4—C3	103.54 (15)	N4'—C3'—C3	109.09 (17)
C5—C4—C3	117.26 (16)	N4'—C3'—H3'1	109.9
O1—C4—H4	109.7	C3—C3'—H3'1	109.9
C5—C4—H4	109.7	N4'—C3'—H3'2	109.9
C3—C4—H4	109.7	C3—C3'—H3'2	109.9
O9—C5—C4	110.29 (16)	H3'1—C3'—H3'2	108.3
O9—C5—C6	103.92 (14)	C5'—N4'—C3'	121.7 (2)
C4—C5—C6	115.15 (17)	C5'—N4'—H4'	116.9 (18)
O9—C5—H5	109.1	C3'—N4'—H4'	120.3 (18)
C4—C5—H5	109.1	O6'—C5'—N4'	125.9 (2)
C6—C5—H5	109.1	O6'—C5'—O7'	125.2 (2)
O7—C6—C5	103.79 (16)	N4'—C5'—O7'	108.9 (2)
O7—C6—H6A	111.0	C5'—O7'—C8'	117.5 (2)
C5—C6—H6A	111.0	O7'—C8'—C1''	106.1 (2)
O7—C6—H6B	111.0	O7'—C8'—H8'1	110.5
C5—C6—H6B	111.0	C1''—C8'—H8'1	110.5
H6A—C6—H6B	109.0	O7'—C8'—H8'2	110.5
C6—O7—C8	105.08 (16)	C1''—C8'—H8'2	110.5
O7—C8—O9	104.38 (16)	H8'1—C8'—H8'2	108.7
O7—C8—C10	108.31 (18)	C2''—C1''—C6''	118.9 (2)
O9—C8—C10	109.48 (17)	C2''—C1''—C8'	120.8 (2)
O7—C8—C11	111.69 (17)	C6''—C1''—C8'	120.2 (3)
O9—C8—C11	109.16 (17)	C3''—C2''—C1''	120.6 (3)
C10—C8—C11	113.42 (18)	C3''—C2''—H2''	119.7
C5—O9—C8	108.74 (14)	C1''—C2''—H2''	119.7
C8—C10—H10A	109.5	C2''—C3''—C4''	120.4 (3)
C8—C10—H10B	109.5	C2''—C3''—H3''	119.8
H10A—C10—H10B	109.5	C4''—C3''—H3''	119.8
C8—C10—H10C	109.5	C5''—C4''—C3''	119.4 (3)
H10A—C10—H10C	109.5	C5''—C4''—H4''	120.3
H10B—C10—H10C	109.5	C3''—C4''—H4''	120.3
C8—C11—H11A	109.5	C4''—C5''—C6''	120.8 (3)
C8—C11—H11B	109.5	C4''—C5''—H5''	119.6
H11A—C11—H11B	109.5	C6''—C5''—H5''	119.6
C8—C11—H11C	109.5	C5''—C6''—C1''	119.9 (3)
H11A—C11—H11C	109.5	C5''—C6''—H6''	120.1
H11B—C11—H11C	109.5	C1''—C6''—H6''	120.1
			
C4—O1—C1—O12	107.54 (18)	C11—C8—O9—C5	−95.99 (19)
C4—O1—C1—C2	−7.1 (2)	O1—C1—O12—C13	−111.56 (19)
O12—C1—C2—O14	−20.6 (2)	C2—C1—O12—C13	4.0 (2)
O1—C1—C2—O14	98.33 (18)	C1—O12—C13—O14	14.4 (2)
O12—C1—C2—C3	−136.12 (17)	C1—O12—C13—C16	131.58 (19)
O1—C1—C2—C3	−17.2 (2)	C1—O12—C13—C15	−104.5 (2)
O14—C2—C3—C3'	49.2 (2)	C3—C2—O14—C13	140.31 (17)
C1—C2—C3—C3'	159.10 (16)	C1—C2—O14—C13	29.82 (19)
O14—C2—C3—C4	−77.1 (2)	O12—C13—O14—C2	−28.1 (2)
C1—C2—C3—C4	32.8 (2)	C16—C13—O14—C2	−144.9 (2)
C1—O1—C4—C5	152.58 (16)	C15—C13—O14—C2	89.6 (2)
C1—O1—C4—C3	28.2 (2)	C2—C3—C3'—N4'	65.0 (2)
C2—C3—C4—O1	−37.23 (19)	C4—C3—C3'—N4'	−176.82 (16)
C3'—C3—C4—O1	−162.69 (16)	C3—C3'—N4'—C5'	−165.57 (19)
C2—C3—C4—C5	−154.43 (17)	C3'—N4'—C5'—O6'	3.5 (4)
C3'—C3—C4—C5	80.1 (2)	C3'—N4'—C5'—O7'	−177.68 (18)
O1—C4—C5—O9	−67.04 (19)	O6'—C5'—O7'—C8'	0.7 (3)
C3—C4—C5—O9	48.4 (2)	N4'—C5'—O7'—C8'	−178.11 (19)
O1—C4—C5—C6	175.77 (16)	C5'—O7'—C8'—C1''	153.0 (2)
C3—C4—C5—C6	−68.8 (2)	O7'—C8'—C1''—C2''	67.3 (3)
O9—C5—C6—O7	−20.8 (2)	O7'—C8'—C1''—C6''	−109.6 (3)
C4—C5—C6—O7	99.97 (19)	C6''—C1''—C2''—C3''	0.9 (4)
C5—C6—O7—C8	35.75 (19)	C8'—C1''—C2''—C3''	−176.1 (2)
C6—O7—C8—O9	−37.11 (19)	C1''—C2''—C3''—C4''	−0.5 (4)
C6—O7—C8—C10	−153.69 (17)	C2''—C3''—C4''—C5''	−0.3 (4)
C6—O7—C8—C11	80.7 (2)	C3''—C4''—C5''—C6''	0.7 (4)
C4—C5—O9—C8	−125.60 (16)	C4''—C5''—C6''—C1''	−0.3 (4)
C6—C5—O9—C8	−1.7 (2)	C2''—C1''—C6''—C5''	−0.5 (4)
O7—C8—O9—C5	23.5 (2)	C8'—C1''—C6''—C5''	176.5 (2)
C10—C8—O9—C5	139.31 (17)		

### Hydrogen-bond geometry (Å, º)

**Table:**

*D*—H···*A*	*D*—H	H···*A*	*D*···*A*	*D*—H···*A*
N4′—H4′···O6′^i^	0.80 (3)	2.51 (3)	3.295 (3)	167 (2)
C6—H6*B*···O9^ii^	0.97	2.32	3.184 (3)	141

Symmetry codes: (i) *x*, *y*+1, *z*; (ii) *x*, *y*−1, *z*.
